# Diagnostic Biomarkers of Microvascular Complications in Children and Adolescents with Type 1 Diabetes Mellitus—An Updated Review

**DOI:** 10.3390/pediatric16030064

**Published:** 2024-09-05

**Authors:** Naser A. Alsharairi

**Affiliations:** Heart, Mind and Body Research Group, Griffith University, Gold Coast, QLD 4222, Australia; naser.alsharairi@gmail.com

**Keywords:** type 1 diabetes mellitus, microvascular complications, biomarkers, children, adolescents

## Abstract

Type 1 diabetes mellitus (T1DM) is regarded as the most chronic autoimmune disease affecting children and adolescents that results from a destruction of pancreatic β-cell and leads to insulin insufficiency and persistent hyperglycemia (HG). Children and adolescents with T1DM are at an increased risk of developing microvascular complications, including diabetic nephropathy (DNE), diabetic retinopathy (DR), and diabetic neuropathy (DNU). The risk factors and prevalence of these complications differ greatly in pediatric studies. Screening for T1DM microvascular complications undergoes different stages and it is recommended to identify early symptoms and clinical signs. The identification of biomarkers in T1DM microvascular complications is needed to provide optimal treatment. Despite several studies on early biomarkers for DNE in children, the potential biomarkers for predicting DR and DNU have not been completely illustrated. This review fills this gap by identifying biomarkers of T1DM microvascular complications in children and adolescents through searches in the PubMed/Medline database.

## 1. Introduction

Type 1 diabetes mellitus (T1DM), also termed autoimmune diabetes, presents as a major chronic disease characterized by insulin deficiency and hyperglycemia (HG), and results from islet autoantibodies and pancreatic β-cell abnormalities [1]. T1DM most commonly occurs during childhood, and it can also develop during adulthood [2], though the incidence peak appears in early puberty (12–14 years) and between the ages of four to six [3,4]. The International Diabetes Federation Diabetes Atlas estimated the number of children with T1DM and reported that the prevalence rates were higher for under 20 years than for under 15 years (1,110,100 vs. 600,900 cases) [5]. Furthermore, higher incidence rates were reported in children under 20 years (149.5/1000/year) compared to those under 15 years (108.3/1000/year) [6]. T1DM in children and adolescents is mostly of uncertain etiology, but genetic predisposition and environmental triggers (enterovirus infections, respiratory tract infections, obesity, cow’s milk protein intake, high gluten intake, disordered eating, unhealthy eating habits, lower microbial diversity, and stressful life events) are thought to play a crucial role in the pathogenesis [7,8,9].

T1DM is diagnosed in children and adolescents based on three criteria: glycated/glycosylated hemoglobin A1c (HbA1c) (48 mmol/mol or ≥6.5% increase in HbA1c), fasting blood glucose (FBG) (7 mmol/L or ≥126 mg/dL), and 2 h plasma glucose (2 h PG) (11.1 mmol/L or ≥200 mg/dL) [10]. T1DM in children and adolescents presents with symptoms such as weight loss, polyuria, polydipsia, and polyphagia [11,12,13]. Children and adolescents with T1DM are at an increased risk of common comorbidities (e.g., diabetic ketoacidosis, hypoglycemia, dyslipidemia, hypertension) and diabetic complications, including microvascular and macrovascular diseases [14,15,16,17].

T1DM microvascular complications in children and adolescents are represented by diabetic nephropathy (DNE), diabetic retinopathy (DR), and diabetic neuropathy (DNU). The presence of microalbuminuria (MAU) (30–299 mg/day) and a decline in the estimated glomerular filtration rate (eGFR) (<60 mL/min/1.73 m^2^) serve as early indicators of DNE [18,19,20,21]. Screening for DNE begins in children aged 12 years with a T1DM duration of >5 years using a random urine albumin-to-creatinine ratio (ACR) [14,22]. DNE prevalence among children and adolescents is rising, and the risk factors associated with this increase are MAU, high blood pressure, obesity, poor glycemic control, low birth weight, and dyslipidemia [19,23]. Several DNE biomarkers are classified according to their ability to detect oxidative stress (OS), inflammation, and tubular/glomerular injuries [18,24].

Children aged 15 years with a T1DM duration of >5 years are screened for DR using digital fundus photography, indirect slit-lamp fundoscopy, or direct ophthalmoscopy [14,22]. The prevalence rates of DR among children (aged < 21 years) are low (<6%) [25,26,27] and reported to be associated with abnormal eGFR, systolic blood pressures, and higher HbA1c [25,26,27,28,29,30]. DNU screening using quantitative sensory/autonomic function testing commences 5 years after diagnosis in children greater than 15 years of age with chronically poor metabolic control [14,22]. The prevalence of DNU among children and adolescents varies substantially based on various screening tests [31]. Obesity, longer diabetes duration, and increased low-density lipoprotein (LDL), triglycerides, cholesterol, as well as diastolic blood pressure, have been reported to correlate with the prevalence of DNU [23,31,32].

A more recent review has largely described early biomarkers of DNE in children [19]. However, no comprehensive review to date has identified the potential biomarkers that are specific to all T1DM microvascular complications. Understanding the biomarkers of T1DM microvascular complications in children and adolescents, particularly those related to inflammation and tubular/glomerular dysfunction, could contribute to the development of novel therapeutics. Thus, the aim of this review is to identify T1DM microvascular complication-related biomarkers in children and adolescents.

## 2. Methods

A literature search of the PubMed/Medline database up to August 2024 was carried out using a combination of the following keywords: “DNE”, “DNU”, “DR”, “T1DM”, “biomarkers”, “children”, and “adolescents”. Studies were included if they reported in English and focused on biomarkers of one or more T1DM microvascular complications in children and adolescents aged ≤ 19 years. Studies focused on biomarkers of microvascular complications in type 2 diabetes mellitus (T2DM) were excluded. The search identified 196 records, of which 34 original articles were considered for possible inclusion, and 162 irrelevant publications were removed.

## 3. Biomarkers of Diabetic Nephropathy

The existing literature point to a large number of studies that have focused on the role of kidney injury (tubular), endothelial dysfunction, microRNAs, OS, and inflammation biomarkers in predicting DNE in children and adolescents with T1DM.

### 3.1. Kidney Injury Biomarkers

Urinary excretion of the retinol-binding protein (RBP) and n-acetyl-β-glucosaminidase (β-NAG) were found to be increased in children with or without MAU compared to healthy children and to be associated with higher mean HbA1c percentages (11.3%). These results suggest that these biomarkers may explain the increased risk of developing DNE in children with T1DM [33]. Increased urinary β-NAG levels were correlated with greater increases in the urinary albumin/creatinine ratio (UCAR) (6.2 mg/gm), serum creatinine (Cr) (0.82 mg/dL), mean HbA1c values (8.8%), and cholesterol (165.4 mg/dL) in children with MAU compared to normoalbuminuria (NAU)/healthy children. This indicates that β-NAG is a useful biomarker for assessing renal tubular injury in children with T1DM and for the early detection of DNE [34]. The neutrophil gelatinase-associated lipocalin (NGAL) values both in urine and serum were elevated in children with T1DM compared to healthy children and were related to the albumin excretion rate (AER) (>30 mg–300 mg/24 h). However, urinary and serum NGAL levels were not associated with HbA1c and eGFR [35]. Urinary NGAL and the NGAL/Cr ratio were reported in higher levels in children with or without MAU compared to healthy counterparts. NGAL and the NGAL/Cr ratio might be used as tubular injury biomarkers to predict DNE in clinical practice [36].

Children with MAU showed higher levels of cystatin-C (sCysC), cyclophilin-A (uCypA), and the uCypA/Cr ratio (uCypA/Cr) in serum and urine compared to healthy children and those with NAU. These biomarkers were increased as a result of decreased high-density lipoprotein (HDL) (46.8 mg/dL), eGFR-Cr (100.7 mL/min/1.73 m^2^), and increased LDL (90.4 mg/dL), mean HbA1c values (7.8%), and UCAR (90 mg/gm). These diagnostic biomarkers may allow for the early detection of DNE in children with T1DM, even before MAU occurs [37]. Children with T1DM who developed MAU had higher liver-type fatty acid-binding protein (L-FABP) levels than in NAU and healthy children. L-FABP was also reported to be a biomarker of increase in mean HbA1c values (>8%) and serum and urinary chitinase-3-like protein 1 (YKL-40) levels (23.8 ng/mL). Increased levels of these biomarkers may reflect tubular injury in the early detection of DNE in children with T1DM, even in the NAU state [38].

Higher levels of urinary NGAL were correlated with increased mean HbA1c values (8.3%) in children with MAU compared to NAU and healthy children. Increased urinary NGAL values were also found in NAU children, even with no signs of glomerular damage. This indicates that NGAL can be used as a tubular injury biomarker in both MAU and NAU children [39]. Children with T1DM had higher levels of NGAL in extracellular vesicle fractions (S and E) than their healthy counterparts. In addition, a positive association between NGAL-E and the median levels of HbA1c (8.8%) 1 year prior to enrollment has been reported in T1DM children. Thus, NGAL may act as an early predictive biomarker of DNE progression in children with T1DM [40]. Cross-sectional evidence shows that higher serum and urine levels of sCysC, urinary kidney injury molecule-1 (KIM-1), and renal resistance (RI) were associated with an increased risk of DNE in T1DM children [41]. Another cross-sectional study in children with T1DM has shown evidence of DNE and identified increased serum sCysC, Cr, and decreased eGFR levels. In addition, serum sCysC was associated with higher median levels of HbA1c (8%; 63.9 mmol/mol 33.3;194) and lower HDL levels (1.4 mmol/L). Therefore, sCysC might be useful as a potential tubular injury biomarker for the assessment of DNE [42].

Compared to their healthy counterparts, children with T1DM, despite having normal MAU, had higher urinary tubular injury and OS biomarkers, including pentosidine/Cr and NGAL/Cr, which were associated with urine microalbumin/Cr (36 mcg/g) [43]. A longitudinal study showed that sCysC and NGAL levels were elevated in children with T1DM compared to their healthy counterparts after a 12–15-month follow-up. These biomarkers were associated with a decreased mean eGFR value and increased both serum Cr and systolic arterial pressure over time. Increased sCysC and NGAL levels may indicate the progress of early DNE in children with T1DM over time [44].

### 3.2. Inflammatory Biomarkers

Transforming growth factor-β1 (TGF-β1), as a potential inflammatory biomarker for DNE, was reported to be higher in T1DM children with a specificity and sensitivity of 100% compared to healthy children [45]. The mRNA of inflammatory genes, including a toll-like receptor (TLR2/4), nuclear factor kappa B (NF-κB), myeloid differentiation primary response 88 (MYD88), membrane cofactor protein 1/chemokine (C-C motif) ligand 2 (MCP1/CCL2), and interleukin 18 (IL-18), have been detected in higher levels in T1DM than normoglycemic children. These genes may lead to an increase in tubular injury and consequently, DNE in children with T1DM [46]. In a retrospective cohort study, T1DM children with DNE compared to those without demonstrated a higher systemic immune-inflammatory index, including the platelet/lymphocyte ratio (PLR), the neutrophil/lymphocyte ratio (NLR), the systemic inflammation response index (SIRI), and the systemic immune-inflammatory index (SII). Children with DNE also showed increased levels of MAU (47.3 vs. 10 μg/min), HbA1C (9.1% vs. 7.7%), platelets (280 vs. 262 10^3^/μL), and cholesterol (167.5 vs. 150 mg/dL) compared to those without [47].

### 3.3. Endothelial Dysfunction Biomarkers

A case–control study provides evidence that the epidermal growth factor (EGF) may increase the risk of DNE in children. T1DM children showed decreased urinary levels of the EGF compared to their healthy counterparts, which were associated with eGFR decline (<30 ng/mg) and MAU lower than 8.5 mg/g [48]. One study suggests that leucine-rich α-2 glycoprotein 1 (LRG1) may be an early biomarker of DNE progression in T1DM children. A 0.282 μg/mL increment in plasma levels of LRG1 was found to be associated with a 1% decrease in eGFR [49]. A cross-sectional study indicates that angiopoietin-2 (Angpt-2) is considered a potential biomarker for the detection of DNE in children with T1DM prior to albuminuria occurrence. Increased serum Angpt-2 levels were reported in children with T1DM compared to their healthy counterparts and those with NAU. Higher serum Angpt-2 levels were also found to be associated with increased levels of UACR (72.8 mg/g Cr), non-HDL-C (135.5 mg/dL), LDL (135.5 mg/dL), and mean HbA1c percentages (9.3%) [50].

### 3.4. MicroRNAs and Other Biomarkers

In a study based on a cross-sectional approach, miR-377 and miR-216a served as potential diagnostic biomarkers for DNE in children with MAU compared to NAU and healthy children. The expression of miR-377 is elevated as a result of increased levels of UACR (78.3 mg/g Cr), carotid intimal thickness (CIMT) (0.67 mm), and mean HbA1C percentages (10.4%) [51]. More cross-sectional evidence indicates the upregulation and/or downregulation of miRNA expression in children with DNE than those without. In children with DNE, the expression of miR-216a was inversely associated with Cr and positively associated with eGFR, whereas the expression of miR-21 was positively associated with urinary sCysC and negatively associated with eGFR [52]. Other evidence has shown that the overexpression of miR-192 and the decline in serum Klotho (KL), a regulator of the fibroblast growth factor, were implicated in DNE. In children with T1DM, serum levels of miR-192 were associated with increased malondialdehyde (MDA) and IL-6, whereas low serum levels of KL were associated with higher mean HbA1c percentages at the time of evaluation (8.3%) and over two years (8%) [53]. Midkine (MK) serves as a potential biomarker for the early detection of DNE. Serum MK levels were reported to be higher in children with MAU and NAU than in healthy children and were related to higher mean HbA1c percentages (9.2%), ACR levels (218.6 mg/g Cr), and high-sensitivity C-reactive protein (hs-CRP) (6.5 mg/L) [54].

Studies highlighting the biomarkers of DNE in children with T1DM are summarized in Table 1.

## 4. Biomarkers of Diabetic Retinopathy

A few studies to date have shown evidence of inflammatory and anti-inflammatory biomarkers related to the occurrence of DR in T1DM children and adolescents.

### 4.1. Inflammatory and Anti-Inflammatory Biomarkers

In one case–control study, high serum levels of inflammatory cytokines, including tumor necrosis factor α (TNF-α), IL-6, and IL-12 were detected in children with nonproliferative DR compared to those without and healthy children. TNF-α was the most reported biomarker that caused damage to the eye fundus in children with DR [55]. In another study, high serum TGF-β1 levels predict the occurrence of DR in children with T1DM. Children with nonproliferative DR had higher levels of TGF-β1 than those without and their healthy counterparts [56]. A study suggests that serum adiponectin levels can be used as an anti-inflammatory biomarker for the early detection of DR. Serum adiponectin levels were reported to be lower in children with DR than their healthy counterparts. No association was reported between diabetes duration and the serum levels of IL-6, TNF-α, leptin, and adiponectin. The serum levels of IL-6 and TNF-α were not different in children with and without DR. The study concludes that low serum adiponectin levels in children with DR should be closely monitored [57].

### 4.2. Other Biomarkers

The other biomarker related to DR was insulin-like growth factor I (IGF-I). Serum IGF-I levels were found to be lower in children with T1DM than their healthy counterparts and were inversely associated with mean levels of HbA1c (6.7–11.3%; 50–100 mmol/mol) over a period of 6 years. Low serum IGF-I levels were also associated with preproliferative DR progression over time [58].

A summary of DR-related biomarkers in children with T1DM is shown in Table 2.

## 5. Biomarkers of Diabetic Neuropathy

A few pediatric studies have focused on early biomarkers of inflammation and genetic biomarkers and microRNAs in DNU.

### 5.1. Inflammatory Biomarkers

In a study of children with DNU compared to those without, high serum levels of inflammatory neopterin were associated with increased latency in ulnar and tibial nerves, hs-CRP (3.8 mg/L), and mean HbA1c levels (10.3%) [59]. Another study has reported increased serum levels of inflammatory biomarkers in children with T1DM and large fiber neuropathy compared to those with different types of neuropathies and healthy children, with a negative association with nervus tibialis conduction velocity [60].

### 5.2. MicroRNAs and Genetic Biomarkers

Genetic variants of T1DM are involved in predicting the early occurrence of neurodegenerative signs in DNU as indicated by positive associations of MIR146A (rs2910164) and MIR128A (rs11888095) polymorphisms with higher values of minimum rim width and in vivo confocal microscopy parameters [61]. Evidence of the association between CYBA polymorphism rs4673 and DNU was reported in children with T1DM. No association was reported between DNU, polymorphism rs1041740 in the SOD1 gene, and polymorphism rs1001179 in the CAT gene [62].

Table 3 shows an overview of DNU-related biomarkers in children with T1DM.

## 6. Biomarkers of Combined Diabetic Microvascular Complications

The limited number of studies available have demonstrated that in children and adolescents with T1DM, biomarkers of inflammation, OS, and anti-angiogenesis are associated with the risk of one or more diabetic complications.

### 6.1. Inflammatory and Oxidative Stress Biomarkers

A study has shown that TGF-β1 is a biomarker of microangiopathy development in T1DM children with a diabetes duration of 10 years. Serum TGF-β1 has been reported in higher levels in children with DNE and DR compared to those without [63]. Another study showed increased levels of OS biomarkers in children with microangiopathy (DNE, DNU, and DR) compared to those without. These biomarkers include myeloperoxidase (MPO) and N-terminal-pro B-type natriuretic peptide (NT-proBNP), which were associated with higher levels of HbA1c (9.6%), total cholesterol (191 mg/dL), and diastolic blood pressure (77 mmHg) [64].

### 6.2. Anti-Angiogenesis and Other Biomarkers

Kallistatin, a novel inhibitor of angiogenesis, was found in higher levels in the serum of children with microvascular complications (DNE, DNU, and DR) than those without and healthy children. Kallistatin was also associated with higher levels of FBG (190.5 mg/dL), systolic and diastolic blood pressure (112.3/80 mmHg), HbA1c (10.7%), total cholesterol (231.9 mg/dL), triglycerides (197.4 mg/dL), LDL (162.9 mg/dL), UACR (168.2 mg/g Cr), hs-CRP (4.3 mg/L), and right and left CIMT (0.69/0.72 mm). Increased kallistatin levels reflect the presence of disturbed angiogenesis, which contributes to microvascular complications in children with T1DM [65]. A study that aimed to assess the relationship between serum advanced glycation end product (AGE) levels and microvascular complications in T1DM children found that serum AGE levels were associated with DNE, but not with DNU and DR. Higher serum AGE levels were also associated with urinary ACR in children with microvascular complications compared to those without [66].

Table 4 presents the biomarkers of combined diabetic complications in children with T1DM.

**Figure 1:**
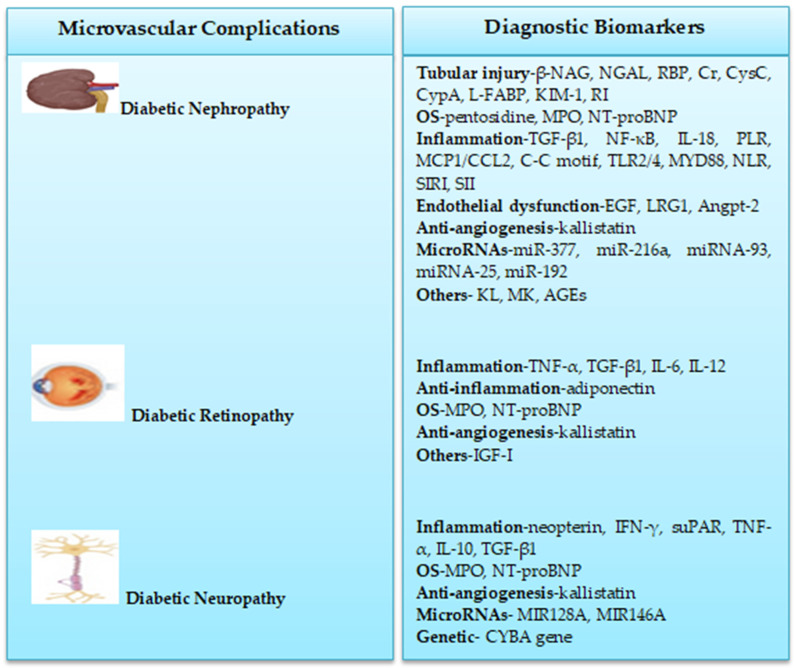
Biomarkers of T1DM microvascular complications in children and adolescents.

Figure 1 shows a summary of biomarkers for detecting microvascular complications in children and adolescents with T1DM. These biomarkers with further robust validation might be used to guide T1DM diagnosis in clinical practice.

## 7. Conclusions

T1DM is the most frequent autoimmune disease in children despite a rise in the incidence rate of this type among adults. T1DM is associated with developing microvascular complications, including DNE, DR, and DNU. Delayed diagnosis in T1DM increases the risk of developing microvascular complications. Screening for T1DM microvascular complications in children and adolescents is necessary to aid in early detection and targeted treatments. There is a need to facilitate early T1DM biomarker testing for efficient clinical trials that aid in developing novel therapeutic options, particularly in children with microvascular complications.

The urinary and serum levels of tubular injury, endothelial dysfunction, OS, and inflammatory biomarkers have been found to be higher in children and adolescents with DNE when compared to healthy children. T1DM resulted in increased levels of RBP, β-NAG, NGAL, Cr, CysC, L-FABP, KIM-1, and RI, which have been identified as tubular injury biomarkers in diagnosing DNE progression. Levels of EGF, LRG1, and Angpt-2, evaluated as endothelial dysfunction biomarkers, are increased in DNE children and adolescents. Higher pentosidine levels predict the early onset of DNE. Inflammatory biomarkers, including TGF-β1, TLR2/4, NF-κB, MCP1/CCL2, IL-18, PLR, NLR, SIRI, and SII could be significant predictors of DNE. Higher levels of miR-377, miRNA-377, miRNA-93, MK, and lower levels of miR-216a, miRNA-25, and KL predict the development of DNE.

Results from a few studies showed that inflammatory and anti-inflammatory biomarkers predict DR development. Children and adolescents with DR demonstrated higher serum levels of TNF-α, IL-6, IL-12, and TGF-β1, and lower levels of adiponectin compared to their healthy counterparts. Lower serum levels of IGF-I could be a potential biomarker for predicting DR. Evidence from a few studies suggests an increase in the serum levels of inflammatory biomarkers, including neopterin, IFN-γ, suPAR, TNF-α, and IL-10 in children and adolescents with DNU compared to their healthy counterparts. Polymorphisms in MIR128A and MIR146A, as well as rs4673 in the CYBA gene, are associated with the early development of DNU in children and adolescents.

Limited evidence showed that children with microangiopathy (DNE, DNU, and DR) had higher serum levels of TGF-β1, MPO, and NT-proBNP, which are used as biomarkers of inflammation and OS, than those without microangiopathy. The serum kallistatin level, identified as an anti-angiogenesis biomarker, was found to be higher in children with microangiopathy compared to those without. Serum levels of AGEs were increased in children with DNE, but not in those with DNU and DR.

Higher levels of diagnostic biomarkers have been shown to be associated with increased levels of AER, LDL, HbA1c, UCAR, Cr, CIMT, and total cholesterol, and decreased levels of eGFR and HDL. The major limitations of all studies include a small sample size and the cross-sectional or case–control design used, which do not allow for the evaluation of the changes in biomarkers of T1DM microvascular complications over time. Further studies with a large sample size are warranted to establish the role of these biomarkers in the pathogenesis of DNE, DNU, and DR. More studies are also required to elucidate the role of microRNAs and genetic variants of T1DM as potential biomarkers in predicting DNE and DNU. Urinary and serum biomarker panels in the prediction of T1DM microvascular complications need validation on longitudinal studies for clinical use.

## Figures and Tables

**Table 1:** Biomarkers of diabetic nephropathy in children and adolescents with T1DM.

Study Design	Study Population	Biomarkers Classification	Biomarkers and Outcomes	Ref.
Case–control study	Total sample = 99 T1DM cases = 59 (MAU = 11; NAU = 48) Healthy controls = 40 Age = 9–19 years	Tubular injury	Higher urinary loss of RBP and β-NAG in diabetic children with MAU (406 µg/dL and 11.16 Ug/Cr) than those with NAU (386.13 µg/dL and 6.88 Ug/Cr) and healthy controls (151.8 µg/dL and 3.8 Ug/Cr). Children with high baseline urinary RBP/β-NAG were assessed every 6 months	[33]
Case–control study	Total sample = 90 T1DM cases = 60 (MAU = 30; NAU = 30) Healthy controls = 30 Age = ~10 years	Tubular injury	Children with MAU had higher urinary β-NAG levels than NAU and healthy children	[34]
Case–control study	Total sample = 37 T1DM cases with DNE = 22Healthy controls = 15 Age = ~13 years	Tubular injury	Higher serum NGAL (sNGAL) and urinary NGAL (uNGAL) levels were detected in children with T1DM compared to healthy controls (sNGAL = 867 ng/mL vs. 655 ng/mL; uNGAL = 420 ng/mL vs. 156 ng/mL)	[35]
Case–control study	Total sample = 111 T1DM cases (MAU and NAU) = 76 Healthy controls = 35 Age = ~12 years	Tubular injury	Higher uNGAL and uNGAL/Cr levels were reported in children with MAU (145 ng/mL and 104 ng/mg) and NAU (92 ng/mL and 121 ng/mg) compared to healthy controls (21 ng/mL and 32 ng/mg)	[36]
Case–control study	Total sample = 90 T1DM cases = 60 (MAU = 31; NAU = 29) Healthy controls = 30 Age = ~13 years	Tubular injury	Higher serum CysC (0.84 mg/dL) and urinary CypA (26.9 ng/mL) and CypA/Cr ratio (0.38 ug/gm) were reported in children with MAU than those with NAU (CysC = 0.69 mg/dL; CypA = 17.85 ng/mL; CypA/Cr ratio = 0.22 ug/gm) and healthy controls (CysC = 0.53 mg/dL; CypA = 15.65 ng/mL; CypA/Cr ratio = 0.19 ug/gm)	[37]
Case–control study	Total sample = 61 T1DM cases = 29 (MAU = 3; NAU = 20) Healthy controls = 32 Age = ~16 years	Tubular injury	Urinary L-FABP/Cr levels were higher in children with MAU (9.8 ng/mg) than those with NAU (6.3 ng/mg) and healthy controls (2.9 ng/mg)	[38]
Cross-sectional study	Total sample = 68 T1DM children = 50 (MAU = 12; NAU = 38) Healthy children = 18 Age = ~14 years	Tubular injury	Children with MAU demonstrated increased uNGAL (39.1 ng/mL) compared to those with NAU (15.6 ng/mL) and healthy controls (5.6 ng/mL)	[39]
Cross-sectional study	Total sample = 49 T1DM children = 34 Healthy children = 15 Age = ~14 years	Tubular injury	Higher uNGAL levels both in S and E fractions were detected in T1DM children than in healthy counterparts	[40]
Cross-sectional study	Total sample = 75 children with T1DM Age = 10–18 years	Tubular injury	Increased serum/urinary sCysC, KIM-1, and RI levels in T1DM children	[41]
Cross-sectional study	Total sample = 779 children with T1DM Age = ~16 years	Tubular injury	Increased serum sCysC and Cr, and decreased eGFR levels in T1DM children	[42]
Cross-sectional study	Total sample = 31 T1DM children = 21 Healthy children = 10 Age = ~16 years	Tubular injury and OS	Higher uNGAL/Cr and pentosidine/Cr were associated with urine microalbumin/Cr	[43]
Prospective cohort study	Total sample = 105 T1DM children = 56 Healthy children = 49 12–15-month follow-up period Age = ~13 years	Tubular injury	Increased serum NGAL and sCysC in T1DM children compared to healthy counterparts over time	[44]
Cross-sectional study	Total sample = 100 T1DM children with DNE = 50 Healthy children = 50 Age = ~10 years	Inflammation	Higher uTGF-β1 levels were reported in T1DM children than in healthy counterparts	[45]
Cross-sectional study	Total sample = 98 T1DM children = 49 Normoglycemic children = 49 Age = ≤19 years	Inflammation	Higher mRNA levels of inflammatory genes were observed in T1DM compared to healthy controls. These genes may lead to DNE progression in children with T1DM	[46]
Retrospective cohort study	Total sample = 100 T1DM with DNE = 34 T1DM without DNE = 66 Age = ~12 years	Inflammation	Increased inflammatory markers, including PLR, NLR, SIRI, and SII acting as early predictors of DNE in T1DM children	[47]
Case–control study	Total sample = 198 T1DM cases = 158 Healthy controls = 40 Age = ~13 years	Endothelial dysfunction	Children with T1DM had lower uEGF levels than healthy controls (46.5 vs. 86.3 ng/mL). Thus, uEGF is considered a potential biomarker of DNE in children with T1DM.	[48]
Cross-sectional study	Total sample = 72 children with T1DM Age = ≤19 years	Endothelial dysfunction	Higher plasma levels LRG1 were detected in T1DM children and associated with sCysC-based eGFR decline. LRG1 is therefore considered as an early biomarker of DNE progression	[49]
Cross-sectional study	Total sample = 90 T1DM children = 60 (MAU = 30; non-albuminuric = 30) Healthy children = 30 Age = ~13 years	Endothelial dysfunction	Higher serum Angpt-2 levels were reported in MAU group than other groups (MAU = 148 ng/L; non-albuminuric = 125.3 ng/L; healthy controls = 90.5 ng/L). Therefore, Angpt-2 acts as a useful diagnostic biomarker for DNE in children with T1DM	[50]
Cross-sectional study	Total sample = 100 T1DM cases = (MAU = 24 with DNE; NAU without DNE = 26) Healthy controls = 50 Age = ~14 years	MicroRNAs	Children with MAU demonstrated higher umiR-377 and lower umiR-216a expression compared to other groups	[51]
Cross-sectional study	Total sample = 70 T1DM children (with DNE = 45; without DNE = 25) Age = ≤18 years	MicroRNAs	Children with DNE compared to those without demonstrated higher expression of miRNA-377 and miRNA-93, and lower expression of miRNA-25	[52]
Cross-sectional study	Total sample = 79 children with T1DM Age = ~16 years	MicroRNAs and others	KL has been considered as a potential biomarker of early DNE in children with T1DM. T1DM children demonstrated increased serum levels of miR-192 and decreased serum levels of KL	[53]
Case–control study	Total sample = 180 T1DM cases = (MAU = 60; NAU = 60) Healthy controls = 60 Age = ~16 years	Others	Higher MK levels have been detected in children with MAU and NAU than in healthy controls (MAU = 1847.2 pg/mL; NAU = 1158.4 pg/mL; 658.3 pg/mL). This suggests that MK is a useful biomarker for the detection of DNE in children with T1DM	[54]

**Table 2:** Biomarkers of diabetic retinopathy in children and adolescents with T1DM.

Study Design	Study Population	Biomarkers Classification	Biomarkers and Outcomes	Ref.
Case–control study	Total sample = 152 T1DM cases = 111 (nonproliferative DR = 21; without DR = 90) Healthy controls = 41 Age = ~15 years	Inflammation	Children with nonproliferative DR had higher serum levels of TNF-α, IL-6, and IL-12 than those without and healthy controls (DR: TNF-α = 1.7 pg/mL, IL-6 = 3.9 pg/mL, IL-12 = 1.8 pg/mL; without DR: TNF-α = 0.6 pg/mL, IL-6 = 1.8 pg/mL, IL-12 = 1.2 pg/mL; healthy controls: TNF-α = 0, IL-6 = 0.5 pg/mL, IL-12 = 0)	[55]
Case–control study	Total sample = 107 T1DM cases = 88 (nonproliferative DR = 31; without DR = 57) Healthy controls = 19 Age = ~15 years	Inflammation	Higher serum TGF-β1 levels were detected in children with nonproliferative DR (1530 pg/mL) than those without (758 pg/mL) and healthy controls (156 pg/mL)	[56]
Case–control study	Total sample = 88 DR cases = 60 Healthy controls = 28 Age = ~12 years	Anti-inflammation	Lower serum levels of adiponectin were detected in children with DR than healthy controls (6.2 vs. 5.0 ng/dL)	[57]
Prospective cohort study	Total sample = 2358 T1DM children = 806 Healthy children = 1552 6-year follow-up period Age = ≤18 years	Others	IGF-I is negatively associated with preproliferative DR. Serum IGF-I levels were lower in children with T1DM compared to those without	[58]

**Table 3:** Biomarkers of diabetic neuropathy in children and adolescents with T1DM.

Study Design	Study Population	Biomarkers Classification	Biomarkers and Outcomes	Ref.
Cross-sectional study	Total sample = 60 Children with DNU = 30 Healthy children = 30 Age = ≤18 years	Inflammation	Children with DNU had higher serum neopterin levels than healthy counterparts (53.5 vs. 17 nmol/L)	[59]
Cross-sectional study	Total sample = 79 adolescents with DNU = 56 (large fiber neuropathy, small fiber neuropathy, autonomic neuropathy, gastrointestinal/enteric neuropathy) Healthy adolescents = 23 Age = 15–18 years	Inflammation	Serum levels of interferon-gamma (IFN-γ), soluble urokinase plasminogen activator receptor (suPAR), TNF-α, and IL-10 were higher in adolescents with T1DM than those without (IFN-γ: T1DM = 5.5 pg/mL, healthy adolescents = 4.2 pg/mL; suPAR: T1DM = 2.5 μg/L, healthy adolescents = 2.1 μg/L; TNF-α: T1DM = 0.8 pg/mL, healthy adolescents = 0.6 pg/mL; IL-10: T1DM = 0.4 pg/mL, healthy adolescents = 0.3 pg/mL) Adolescents with large fiber neuropathy had higher serum levels of TNF-α than other different types of neuropathies (large fiber neuropathy = 0.90 pg/mL, small fiber neuropathy = 0.77 pg/mL, autonomic neuropathy = 0.85 pg/mL, gastrointestinal/enteric neuropathy = 0.84 pg/mL)	[60]
Cross-sectional study	Total sample = 140 children with T1DM Age = 17 years	MicroRNAs	Polymorphisms in MIR128A and MIR146A genes are associated with early signs of DNU	[61]
Cross-sectional study	Total sample = 204 T1DM children = 114 Healthy children = 90 Age = ~16 years	Genetic	DNU was associated with polymorphism rs4673 in the CYBA gene in T1DM children only	[62]

**Table 4:** Biomarkers of combined diabetic complications in children and adolescents with T1DM.

Study Design	Study Population	Biomarkers Classification	Biomarkers and Outcomes	Ref.
Case–control study	Total sample = 135 T1DM cases = 116 (DNE = 11; DR = 8; DNE and DR = 26; without microangiopathy = 71) Healthy controls = 19 Age = ~13 years	Inflammation	Children with microangiopathy demonstrated higher serum levels of TGF-β1 compared to those without and healthy controls	[63]
Case–control study	Total sample = 72 T1DM cases = 50 (with DNE, DR, and/or DNU = 30; without microangiopathy = 20) Healthy controls = 22 Age = ~17 years	OS	Children with microangiopathy showed higher levels of MPO and NT-proBNP compared to those without and healthy controls	[64]
Case–control study	Total sample = 90 T1DM cases = 60 (with DNE, DR, and DNU = 30; without microangiopathy = 30) Healthy controls = 30 Age = ~14 years	Anti-angiogenesis	Higher serum kallistatin levels were detected in microvascular complications group than other groups (with microvascular complications = 9.9 ng/mL; without microvascular complications = 5.0 ng/mL; healthy controls = 1.3 ng/mL)	[65]
Case–control study	Total sample = 84 Children with microangiopathy (DNE, DR, and DNU) = 26 Children without microangiopathy = 58 Age = 9–18 years	Others	Children with DNE had higher serum AGE levels than those with DNU, DR, and healthy children	[66]

## Data Availability

Not applicable.

## Abbreviations

2 h PG	2 h plasma glucose
ACR	Albumin to creatinine ratio
AER	Albumin excretion rate
AGEs	Advanced glycation end products
Angpt-2	Angiopoietin-2
CCL	Chemokine (C-C motif) ligand
CIMT	Carotid intimal thickness
Cr	Creatinine
DNE	Diabetic nephropathy
DNU	Diabetic neuropathy
DR	Diabetic retinopathy
EGF	Epidermal growth factor
eGFR	Estimated glomerular filtration rate
FBG	Fasting blood glucose
HbA1c	Glycosylated hemoglobin A1c
HDL	High density lipoprotein
HG	Hyperglycemia
hs-CRP	High-sensitivity C-reactive protein
IFN-γ	Interferon-gamma
IGF-I	Insulin-like growth factor I
IL	Interleukin
KIM-1	Urinary kidney injury molecule-1
KL	Klotho
LDL	Low density lipoprotein
L-FABP	Liver-type fatty acid binding protein
LRG1	Leucine-rich α-2 glycoprotein 1
MAU	Microalbuminuria
MCP	Membrane cofactor protein
MDA	Malondialdehyde
MK	Midkine
MPO	Myeloperoxidase
MYD88	Myeloid differentiation primary response 88
NAU	Normoalbuminuria
NF-κB	Nuclear factor kappa B
NGAL	Neutrophil-gelatinase-associated lipocalin
NGAL/Cr	NGAL/creatinine ratio
NLR	Neutrophil/lymphocyte ratio
NT-proBNP	N-terminal-pro B-type natriuretic peptide
OS	Oxidative stress
PLR	Platelet/lymphocyte ratio
RBP	Retinol binding protein
RI	Renal resistance
sCysC	Cystatin-C
SII	Systemic immune-inflammatory index
SIRI	Systemic inflammation response index
suPAR	Soluble urokinase plasminogen activator receptor
T1DM	Type 1 diabetes mellitus
T2DM	Type 2 diabetes mellitus
TGF-β1	Transforming growth factor-β1
TLR	Toll-like receptor
TNF-α	Tumor necrosis factor α
UCAR	Urinary albumin/creatinine ratio
uCypA	Cyclophilin-A
uCypA/Cr	uCypA/creatinine ratio
YKL-40	Chitinase-3-like protein 1
β-NAG	N-acetyl-β-glucosaminidase
